# The role of the clinical departments for understanding patient heterogeneity in one-year mortality after a diagnosis of heart failure: A multilevel analysis of individual heterogeneity for profiling provider outcomes

**DOI:** 10.1371/journal.pone.0189050

**Published:** 2017-12-06

**Authors:** Nermin Ghith, Anne Frølich, Juan Merlo

**Affiliations:** 1 Research Unit for Chronic Conditions, Department of Clinical Epidemiology, Bispebjerg and Frederiksberg University Hospital, Copenhagen, Denmark; 2 Unit for Social Epidemiology, Faculty of Medicine, Lund University, Malmö, Sweden; Kaohsiung Medical University Hospital, TAIWAN

## Abstract

**Purpose:**

To evaluate the general contextual effect (GCE) of the hospital department on one-year mortality in Swedish and Danish patients with heart failure (HF) by applying a multilevel analysis of individual heterogeneity.

**Methods:**

Using the Swedish patient register, we obtained data on 36,943 patients who were 45–80 years old and admitted for HF to the hospital between 2007 and 2009. From the Danish Heart Failure Database (DHFD), we obtained data on 12,001 patients with *incident* HF who were 18 years or older and treated at hospitals between June 2010 and June2013. For each year, we applied two-step single and multilevel logistic regression models. We evaluated the general effects of the department by quantifying the intra-class correlation coefficient (ICC) and the increment in the area under the receiver operating characteristic curve (AUC) obtained by adding the random effects of the department in a multilevel logistic regression analysis.

**Results:**

One-year mortality for Danish *incident* HF patients was low in the three audit years (around 11.1% -13.1%) and departments performed homogeneously (ICC ≈1.5% - 3.5%). The discriminatory accuracy of a model including age and gender was rather high (AUC≈ 0.71–0.73) but the increment in AUC after adding the department random effects into these models was only about 0.011–0.022 units in the three years.

One-year mortality in Swedish patients with first hospitalization for heart failure, was relatively higher for 2007–2009 (≈21.3% - 22%) and departments performed homogeneously (ICC ≈ 1.5% - 3%). The discriminatory accuracy of a model including age, gender and patient risk score was rather high (AUC≈ 0.726–0.728) but the increment in AUC after adding the department random effects was only about 0.010–0.017 units in the three years.

**Conclusion:**

Using the DHFD standard benchmark for one-year mortality, Danish departments had a good, homogeneous performance. In reference to literature, Swedish departments had a homogeneous performance and the mortality rates for patients with first hospitalization for heart failure were similar to those reported since 2000. Considering this, if health authorities decide to further reduce mortality rates, a comprehensive quality strategy should focus on all Swedish hospitals. Yet, a complementary assessment for the period after the study period is required to confirm whether department performance is still homogeneous or not to determine the most appropriate action.

## Introduction

Scandinavian countries have a number of clinical and population registers that are used for monitoring citizens’ health status, including patients with heart failure (HF). Concurrently, there are several quality audits and national health schemes using these registers to improve the quality of care [1, 2]. One of the most dominant forms of auditing is benchmarking and profiling hospital performance by analyzing differences between hospital averages of quality indicators such as one-year mortality after HF [3, 4]. Generally, profiling analyses aim to compare medical provider quality of care with standards of performance, benchmarks or overall national rates [2, 5, 6]. Profiling analyses can be used for initial or routine monitoring of processes and outcomes of care, identifying potential outliers (providers with less desired performance) and/or ranking providers [2, 5, 7]. In practice, comprehensive provider profiling is carried out periodically, usually annually. It is assumed that continuous monitoring of outcomes of care is required to control and improve provider performance. Here, the ultimate goal for a number of health authorities is to foster the development of a more homogeneous well performing hospital system. [2] Eventually, there should be minimal heterogeneity in care (small provider variance) coupled with high quality outcomes of care. [2, 8].

We have previously shown that to assess whether this goal is achieved, it is not enough to analyze differences between provider (e.g., hospital) averages of the quality indicator [2, 8]. Rather, it is imperative to use an analytical approach like multilevel regression models that can quantify the share of the total patient *heterogeneity* in the outcome that exists at the provider level [2, 8–12]. A provider ranking or profiling tool needs to be accompanied by measures of the *general contextual effects* like the intra-class correlation coefficient (ICC) and the area under the Receiving Operator Characteristics Curve (AUC) for provider random effects [2, 12, 13].Still, the vast majority of profiling analyses has been based on traditional analytical approaches quantifying differences between hospital averages rather than on the more appropriate multilevel regression analysis (MLRA) of individual patient heterogeneity [2, 12]. Using the ICC [8–10, 14, 15], we can evaluate the general effects of the provider as the share of the patient differences in an outcome that is at the provider level; such information is also fundamental for interpreting the rank of the providers (i.e., league tables) according to their average values for a quality indicator (e.g., one-year year mortality after HF). Furthermore, calculating the AUC for the random effects [2, 12, 13, 16] of the provider (e.g., hospital, clinical department) enables us to determine to what extent knowledge on the healthcare unit where the patients are treated provides accurate information for discriminating the patients who survive from those who do not survive [2]. Here, it is imperative to interpret the ICC and the AUC values together for a meticulous assessment of provider variance [2, 12, 13].

In practice, the general contextual effects of the medical provider could take place at the hospital or the department level where the patients are treated or at both levels [2]. If differences in the patient outcome are conditioned by the general context [8, 12] (e.g., indicated by a *meaningful* size of the ICC) of the level of care, this means that the hospital (department) care is heterogeneous. This indicates that the patient outcome depends on the clinical department where the patient was treated [2, 8]. Such reasoning is supported by literature where a small provider variance would indicate a homogeneous clinical practice for a given level of care [2, 8].

Fundamentally, assessment of provider performance or the general contextual effect through monitoring or profiling analyses depends on the type of the performance indicators (measures of outcomes of care). For example, empty, multilevel regression models could be used for provider profiling in relation to a wide range of process indicators. Processes of care are perceived to be under the provider control, where normally there is no need to account for patient case-mix [17, 18]. In this case, the obtained variance (quantified with the ICC) represents the ceiling of the general contextual effect of the provider level of care. However, in the case of outcomes of care, the obtained department residuals from the empty models are susceptible to be confounded by the patient case-mix [2, 7, 8]. This means that the observed department variance can be due to differences in the patient’s health status at admission, rather than department quality of care. Hence, the obtained between-provider variance might actually represent an overestimate of the true one. In order to obtain a realistic value of the general contextual effect of the provider, the best available information on the patient health status (case-mix) should be included in the profiling models [7].

Development of case-mix adjustment models for profiling analyses is, however, a time and resource-intense activity, as information on patient case-mix is not always available. In several situations, provider performance could be under control which requires only a routine monitoring and not a full profiling of performance. Hence, profiling analyses should only be carried out when there is an indication for it (e.g., a meaningful provider ICC) [2, 19]. Otherwise, either empty multilevel regression models or, even better, multilevel models accounting for basic clinically relevant patient information (e.g., gender and age [20, 21]) could be used first for the initial exploration of between provider variance to identify the ceiling of the general contextual effect. The health authorities can then make informed decisions on the value of the obtained variance; does it require further explanation (partition) in an advanced case-mix profiling analysis (e.g., addition of other clinical indicators on patient health condition); and the need for provider ranking and quality improvement interventions [2, 12, 13, 19].

In view of this background, in this study, we apply two-steps single and multilevel logistic regression analysis of individual heterogeneity [2, 12, 22] to evaluate provider performance (i.e., general contextual effect) at the hospital department level in Sweden and Denmark in relation to patient one-year mortality after heart failure. We use two scenarios (empirical examples) to demonstrate the application of our approach for routine monitoring and profiling analyses. The first empirical case includes a Danish cohort of patients with incident HF. In Denmark, there is a special program including the Danish Heart Failure Database “DHFD” along with an audit commission for monitoring and assessing the quality of care for patients with incident heart failure [1]. Between 2010 and 2013, the oversight body for this audit scheme decided that the standard hospital benchmark for one-year morality rate should be equal to or less than 20% [23]. Hence, Danish hospital performance was annually audited against this benchmark. Applying our approach, we aim to demonstrate that with simple available information on the very hospital department where the patients were treated, as well as their age and sex, it is possible to quantify the ceiling of the general contextual effect in routine monitoring of the performance of the Danish medical provider (i.e., hospital departments). Considering a second scenario with a Swedish cohort of patients with first hospitalization for HF, in a full profiling analysis, the general contextual effect of the departments will be estimated after adjustment for the patient case-mix. The findings from both cases are then used to indicate the required actions or quality interventions.

Considering this approach, two sources of information are required to evaluate the provider (hospital clinical departments) performance: 1) the departments’ overall average of the patient one-year mortality, and 2) the size of the general contextual effect (ICC and AUC).

## Population and methods

### Study population

#### Danish cohort

The Danish Heart Failure Database “DHFD” audit commission records data on incident HF patients who are treated at any hospital in the country. The database audit commission records seven indicators focusing on processes of care, treatments, readmission and one-year mortality rates [1]. To obtain data on mortality, the DHFD audit commission links the information available in the DHFD database to the Citizens Personal Register, identifying patients with a unique personal identification number.

We obtained data from the DHFD on 12,001 patients with incident heart failure defined by the following (International Classification of Diseases ICD-10) codes: **I**11.0, **I**13.0, **I**13.2, **I**42.0, **I**42.6, **I**42.7, **I**42.9, **I**50.0, **I**50.1, and **I**50.9 [1, 23]. The patients were 18 years or older and were newly diagnosed with HF upon hospitalization for the first time (at either an out- or inpatient department) between 21 June 2010 and 30 June 2013 (this period covers three audit years). Often, outpatients were admitted to the hospital earlier for treatment for an acute myocardial infarction, and they developed heart failure while admitted, so after treating their heart condition, they were referred by a cardiologist to the hospital ambulatory care (outpatient) unit to follow a special quality program for incident heart failure patients [1].

#### Swedish cohort

Using Swedish patient register, we analyzed data on patients with a first diagnosis of HF; identified with discharge diagnosis of heart failure (International Classification of Diseases ICD-10 code **I**50). The patients were 45–80 years old and admitted to hospitals in the period between 2007 and 2009. The database has already described in detail elsewhere [2].

As our goal was to evaluate hospitals, we included clinical departments in all hospitals in Sweden (public facilities), but excluded nursing and elderly homes, as well as private rehabilitation facilities. The final dataset included 36, 943 patients within 565 departments from 71 hospitals.

Considering the inclusion criteria for each cohort, it is obvious that the Danish [1] and the Swedish populations of patients differ in severity of illness. Meaning that the patient populations in the Danish and Swedish cohorts are very different and not comparable. The Swedish patients are hospitalized for HF and therefore have a higher absolute risk than the Danish patients. DHFD case finding includes a large amount of hospital outpatients in an effort to identify patients at the onset stage of illness [1].

### Assessment of patient variables

#### Patient outcome

In the Swedish cohort, the study outcome is all-cause mortality within one-year after discharge from the hospital. In the Danish cohort, the study outcome is all-cause mortality within one-year after a first contact with a hospital [23].

In the Danish cohort, following DHFD audit routines, one-year mortality is evaluated for three consecutive audit periods from 21 June 2010 to 30 June 2013 (i.e., audit period 1: 21 June 2010–20 June 2011; audit period 2: 21 June 2011–20 June 2012; audit period 3: 1 July 2012–30 June 2013). We followed the calendar year to carry out annual evaluations for the Swedish cohort one-year mortality outcomes between 2007 and 2009.

#### Patient characteristics

In statistical analyses for both cohorts of patient, gender was included as a dummy variable and age as a continuous variable.

In both cohorts, we allowed for a potential non-linear effect of age on mortality by fitting a quadratic function for age in the models (see below). However, the association was linear, so we kept age as a continuous variable in the final analyses.

In the Swedish cohort, we modelled one-year mortality as a function of previous diseases or patient case-mix (using ICD-10 codes) and obtained the predicted probability (i.e., individual risk score “RS”) following a similar procedure as described elsewhere [2].

### Statistical and epidemiological analyses

To identify the proper level of analysis, in each cohort and for each year, we fit a three-level empty model (patients within departments within hospitals) in an explorative phase. We found a very small hospital variance, so we adopted a two-level regression analyses with patients nested within departments in the main analyses.

In both cohorts, for each year, we developed a two-step single and multilevel logistic regression analysis of individual heterogeneity to model the risk of one-year mortality [2, 12, 13]. We combined information from both steps (see below) to assess the general contextual effect of the department level of care. First, we started with conventional single-level logistic regression model that included the individual patient-level variables; departments were completely omitted. In a subsequent model, we added a random intercept for the department level applying a multilevel logistic regression.

From each model, we calculated the predicted logit and the Area Under the Receiver Operator Characteristics Curve (AUC). The AUC measures the discrimination ability of each model to correctly classify patients with or without the outcome (i.e., mortality within one-year) [24].

In MLRA analyses, we obtained two different measures of the general contextual effects: (i) the intra-class correlation coefficient (ICC) and (ii) the AUC value. We calculated the ICC for the department level according to the latent variable method [15, 25] as

$$\mathrm{ICC}=\frac{\sigma^{2}}{\left(\sigma^{2}+\frac{\pi^{2}}{3}\right)}$$ (1)

Where σ^2^ is the department level variance, and $\frac{\pi^{2}}{3}$ represents the variance of a standard logistic distribution. (*π* here represents the mathematical constant 3.1416) [25].

The ICC indicates the correlation in the underlying propensity of death between two patients randomly picked from the same hospital department. The ICC is expressed as a percentage that goes from zero to 100. An ICC close to zero means that the departments would be similar to random samples taken from the whole patient population with HF. This means that department performance is homogeneous.[2, 8, 12]

In the calculation of the AUC for the MLRA models, the prediction equation includes the random effects (i.e., higher level residuals of departments) as discussed elsewhere [12, 13, 16].

We obtained the Odds Ratio (OR) and 95% confidence intervals (CI) for patient covariates.

#### Step 1. Single-level analysis with patient predictors

The first single-level logistic regression model (model 1) informed us on the association between the individual patient characteristics and mortality. In the Danish cohort, the model included only gender and age. In the Swedish cohort, this model includes gender, age and the patient risk score described elsewhere [2]. In model 1, we also obtained the AUC to estimate the ability of the individual patient level information *alone* to discriminate between the patients who died from those who survived.

#### Step 2. Multilevel analysis: Department general contextual effect

In the next model (model 2), we extended model 1 to include the random intercept of the department in a two-level multilevel logistic regression model. In model 2, we calculated the ICC and the difference between the AUC values for models 2 and 1. In doing so, we aimed to investigate whether knowledge of the department where the patient was treated improved our ability to discriminate between patients who lived from those who died, over and above patient information alone. Here, the AUC evaluates the relevance of the departments for patients’ one-year mortality. This information complements the information obtained by the ICC as a measure of general contextual effects [13, 16]. Any increase in the AUC values in the general contextual effects model 2 compared to model 1 (with only patient predictors) will represent both measurable and immeasurable department factors that could condition the survival of the HF patients.

In both cohorts, we fit multilevel random intercept models assuming that the effects of individual patient characteristics such as gender, age and risk score were the same across departments. However, we also relaxed this assumption by allowing the slopes of regression coefficients to be random at the hospital department level [26]. Since we did not find any conclusive variation in the random slopes, our multilevel models contain only random intercepts.

### Department league tables

From the multilevel models, we obtained the values of the shrunken residuals for the Swedish and the Danish departments and their 95% confidence intervals to rank the departments according to their average one-year mortality. For interpreting the rank, we use the information provided by the ICC in the multilevel model 2, and the AUC change between the single-level regression model 1 and the multilevel regression model 2.

### Models estimation

We used the Restricted Iterative Generalized Least Squares (RIGLS) method to obtain the initial values for the final Markov Chain Monte Carlo (MCMC) estimation method [27]. The variance was estimated as the median and 95% credible intervals of the posterior distribution obtained by the Markov Chain Monte Carlo (MCMC) method [27]. We used the Bayesian Deviance Information Criterion (DIC) as a measure of the goodness of fit of the models [28]. We used the statistical program SPSS version 23 (SPSS Inc., Chicago, IL, USA), and MLwiN version 2.31,the Centre for Multilevel Modeling, University of Bristol for statistical analyses [29].

## Results

### Descriptive statistics

Considering the Danish cohort, Table 1 shows that the total number of incident HF patients was almost the same in the three audit periods. The overall national crude rate of one-year mortality for HF patients decreased from 13.1% in the audit period 1 to 11.1% in the audit period 3. Further, a large number of the Danish patients were treated at outpatient clinics, where the mortality rate in this group (outpatients) was much lower than in inpatients.

**Table 1:** Crude national mortality rates, number of heart failure patients, metrics on gender and age, and hospital departments included in the study.

	Danish cohort (incident heart failure patients)			Swedish cohort (patients with first hospitalization for heart failure)		
	Audit Period 1^a^	Audit Period 2	Audit Period 3	2007	2008	2009
**Average Mortality Rate (%)**	13.1	11.6	11.1	22	21.3	21.5
**Outpatients**^**b**^ **(%)**	53	57.3	60	-	-	-
**Mortality in outpatients**^**b**^ **(%)**	7	6	7	-	-	-
**Mortality in inpatients**^**b**^ **(%)**	20	18	17	-	-	-
**Number of patients**	3995	3980	4026	12366	12644	11933
**Female (%)**	32.7	31.8	31.5	40.7	40.4	40.7
**Age (median)**	72	71.3	70.8	70.6	73	73
**Number of departments**	41	38	42	434	459	459
**Number of hospitals**	32	32	32	71	71	71

^a^ Audit Period 1: 21 June 2010–20 June 2011. Audit Period 2: 21 June 2011–20 June 2012. Audit Period 3: 1 July 2012–30 June 2013.

^b^ Aggregated data obtained from the DHFD annual audit reports.

Considering the Swedish cohort, Table 1 shows that the total number of patients with first hospitalization for HF was almost the same in the three years. The overall national crude rate of one-year mortality for HF was 22%, 21.3%, and 21.5% for the three years, respectively.

In both cohorts and in all years, medium age was relatively similar, and the percentage of females was lower than males, especially in the Danish cohort.

### Measures of association: Individual patient effects

Considering findings from the multilevel models, Table 2 shows measures of association (i.e., fixed effects) for patient covariates.

**Table 2:** Measures of association (fixed effects) obtained by two-level (patients and hospitals departments) multilevel logistic regression modelling one-year mortality for heart failure patients treated in clinical departments. Values are odds ratios (OR) and 95% confidence intervals (CI).

	Danish cohort			Swedish cohort		
	Audit Period 1	Audit Period 2	Audit Period 3	2007	2008	2009
**Male**	Reference			Reference		
Female	1.22 (0.99–1.47)	0.85 (0.68–1.05)	0.92 (0.75–1.14)	0.81 (0.74–0.89)	0.87 (0.8–0.95)	0.91 (0.83–1)
**Age**	1.08 (1.07–1.09)	1.08 (1.07–1.09)	1.07 (1.07–1.09)	1.04 (1.04–1.05)	1.04 (1.03–1.04)	1.04 (1.03–1.04)
Risk score* **(decile groups)**						
1^st^				Reference		
2^nd^				1.68 (1.23–2.19)	1.04 (0.77–1.41)	1.33 (1–1.82)
3^rd^				1.79 (1.34–2.26)	1.45 (1.15–1.86)	1.69 (1.31–2.24)
4^th^				2.18 (1.59–2.92)	1.64 (1.24–2.21)	2.23 (1.63–3.03)
5^th^				2.46 (1.84–3.14)	2.45 (1.94–3.17)	2.54 (1.97–3.38)
6^th^				3.23 (2.46–4.12)	2.73 (2.17–3.56)	3.53 (2.77–4.72)
7^th^				4.12 (3.18–5.24)	3.66 (2.91–4.64)	4.15 (3.28–5.53)
8^th^				5.08 (3.91–6.35)	4.23 (3.41–5.42)	5.39 (4.31–7.03)
9^th^				7.55 (5.92–9.42)	5.89 (4.65–7.46)	6.79 (5.3–8.82)
10^th^				14.03 (10.79–17.41)	11.59 (9.37–14.69)	13.18 (10.38–17.37)

* Risk Score (RS) obtained from a logistic regression including patient case-mix or previous diagnoses (ICD-10) of diseases of the cerebral arteries (I6), arrhythmia (I48-I49), hypertension (I10-I13&I15), ischemic coronary artery disease (I20-I25), varicose (I83), peripheral vascular disease (I74&I80), acute myocardial infarction (I21), other types of heart disease (I3-I5), respiratory diseases (J0-J9), digestive diseases (K0-K9), diabetes (E10-E14), infectious diseases (A0-A9), cancer (C1-D4), lung cancer (C34), chronic diseases of the lower respiratory tract (J4), immunity disorder (D50-D89), mental diseases (F0-F9), and injury (S00-T14).

Audit Period 1: 21 June 2010–20 June 2011. Audit Period 2: 21 June 2011–20 June 2012. Audit Period 3: 1 July 2012–30 June 2013.

In the Danish cohort, as expected, mortality rates increased with age in all models and for all three periods. For audit period 1, females had a higher risk of mortality compared to males. However, these results were reversed over audit periods 2 and 3, as females had a slightly lower risk of mortality. Yet findings on the gender variable were not significant as all confidence intervals included one.

In the Swedish cohort, as expected, the RS was strongly associated with one-year mortality. Independently of the RS, we additionally observed that Swedish females have a lower mortality risk than males. Further, mortality rates increased with age in all models.

### Measures of variance and discriminative accuracy: The general contextual effects of the department

Table 3 shows measures of variance and discrimination along with DIC values for all the models and years.

**Table 3:** Measures of area under the receiver operating characteristic curve (AUC) and measures of variance (random effects) obtained by single-level logistic regression (model 1) and two-level (patients and hospital departments) multilevel logistic regression (models 2) modelling one-year mortality in patients treated in Danish and Swedish departments. Variance measures are expressed as median values and 95% credible intervals (CI).

	**Single-level model 1**^**a**^	**Multilevel model 2**	**Single-level model 1**	**Multilevel model 2**	**Single-level model 1**	**Multilevel model 2**
	**Audit period 1**^**b**^		**Audit period 2**		**Audit period 3**	
** AUC**	0.723 (0.700–0.747)	0.742 (0.719–0.765)	0.727 (0.702–0.752)	0.746 (0.721–0.771)	0.711 (0.686–0.737)	0.722 (0.697–0.747)
Change in AUC	Reference	0.019	Reference	0.022	Reference	0.011
**Variance Measures**						
Department variance		0.115 (0.027–0.281)		0.117 (0.033–0.276)		0.05 (0.006–0.159)
ICC (%)		3.4 (0.8–7.9)		3.5 (1–7.8)		1.5 (0.2–4.6)
**DIC**	2824.3	2801.109	2596.62	2576.723	2572.77	2566.904
** **						
	**Single-level model 1**^**c**^	**Multilevel model 2**	**Single-level model 1**	**Multilevel model 2**	**Single-level model 1**	**Multilevel model 2**
	**2007**		**2008**		**2009**	
** AUC**	0.726 (0.715–0.737)	0.736 (0.726–0.746)	0.727 (0.716–0.737)	0.744 (0.734–0.754)	0.728 (0.717–0.739)	0.740 (0.729–0.75)
Change in AUC	Reference	0.01	Reference	0.017	Reference	0.012
**Variance Measures**						
Department variance		0.050 (0.019–0.084)		0.102 (0.057–0.151)		0.061 (0.020–0.106)
ICC (%)		1.5 (0.6–2.5)		3 (1.7–4.4)		1.8 (0.6–3.1)
**DIC**	11653.469	11627.492	11719.555	11639.717	11112.444	11074.028

^a^ Audit Period 1: 21 June 2010–20 June 2011; Audit Period 2: 21 June 2011–20 June 2012; Audit Period 3: 1 July 2012–30 June 2013.

^b^In the Danish cohort: Model 1: single-level model with gender and age. Model 2: two-level model with gender and age.

^c^In the Swedish cohort: Model 1: single-level model with gender, age and risk score. Model 2: two-level model with gender, age and risk score.

ICC: Intraclass Correlation. PCV: Proportional Change in Variance. DIC: Bayesian Deviance Information Criterion.

In both cohorts, in each year, there was a small department variance. Analogously, the ICC values obtained from the multilevel models with patient information were very small. In the Danish cohort, the ICC values obtained from the multilevel model with gender and age were lowest in the last audit period (i.e., 3.4%, 3.5%, and 1.5% respectively). In the Swedish cohort, the ICC values obtained from the multilevel model with patient gender, age and RS were 1.5%, 3%, and 1.8%, for the three years respectively.

In both cohorts, single-level models with only patient information had rather good AUC values. In the Danish cohort, AUC values obtained from the single-level models were 0.723, 0.727, and 0.711 for the three audit periods, respectively.

Similarly, in the Swedish cohort, single-level model with gender, age and the patient risk score had AUC values of 0.726, 0.727, and 0.728 for the three consecutive years between 2007–2009. However, the good AUC values in the single models were almost unchanged by adding the department random effects in the multilevel models.

Interpreting these findings for the AUC values along with the small ICC values obtained from the MLRA models, indicates that department performance (i.e., general contextual effects) is homogeneous in both Sweden and Denmark.

In both cohorts, in reference to single-level models with only patient information, DIC values improved in the multilevel models which included patient information as well as the random intercepts for the departments.

### Ranking clinical departments–league tables

**Fig 1:**
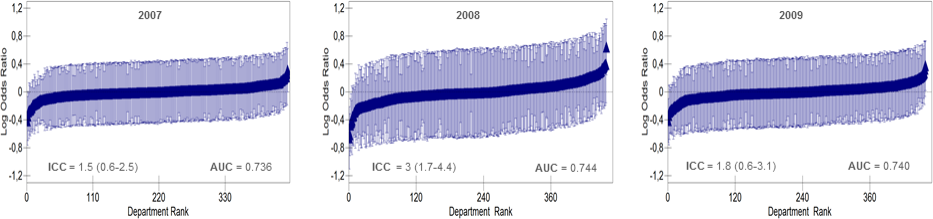
**Ranking of the 565 Swedish departments for the three years (A-C)** according to their one-year mortality after hospitalization for heart failure (2007–2009) using the overall average as reference. Values are logarithm odds ratios (i.e., shrunken residuals) with 95% confidence intervals (vertical lines) adjusted for age, gender and risk score (see model 2 in Table 3). The figure also indicates the values of the departments intra-class correlation coefficients (ICC) for one-year mortality and the AUC.

**Fig 2:**
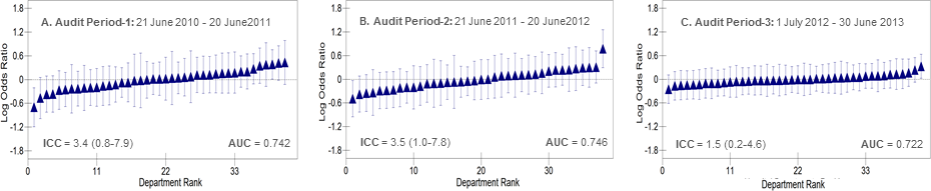
**Ranking of 57 Danish departments for the three audit periods (A-C)** according to their one-year mortality for incident heart failure (21 June 2010–30 June 2013) using the overall average as reference. Values are logarithm odds ratios (i.e., shrunken residuals) with 95% confidence intervals (vertical lines) adjusted for age and gender (see model 2 in Table 3). The figure also indicates the values of the departments intra-class correlation coefficients (ICC) for one-year mortality and the AUC.

Figs 1 and 2 show the ranking of Swedish and Danish departments in the study period, for each cohort and year separately (A-C), according to their average one-year mortality. Values are logarithm odds ratios (i.e., shrunken residuals) with 95% confidence intervals (vertical lines) adjusted for gender and age for the Danish cohort, and adjusted for gender, age and the risk score for the Swedish cohort (see the multilevel models, Table 3). There is a considerable uncertainty in the estimated averages which result in an overlap of the 95% CIs. The figures indicate the values of the small intra department correlation for one-year mortality, which together with the small increase in the AUC in the multilevel models compared to the single models suggests that the departments are homogeneous in performance.

## Discussion

Applying an original approach for multilevel analysis of individual heterogeneity [2, 12, 13], we observed that patient differences in one-year mortality in the Danish cohort (incident HF patients) and the Swedish cohort (first hospitalization for heart failure) did not substantively depend on the clinical department where the patient was treated. That is, mortality after HF was rather homogeneously distributed in both hospital department systems. In other words, mortality after HF did not cluster within hospital departments. In fact, the general contextual effect of the department was very small as expressed by the trivial ICC and the minor change of the AUC when including the department as a random intercept in a MLRA (see Table 3).

*Profiling* or audit analyses based on simple quantification of differences in average mortality between (hospital) departments provide insufficient information on actual performance at this level of care [2, 30]. Such information cannot be used for discriminating patients who will survive or not. Further, what matters most is not the department variance itself but the share of the total individual differences in the propensity of dying that are at the department level [2, 8, 31]. Considering this perspective, the very small department ICC values suggests that clinical departments in each country had a uniform performance.

In the Danish cohort, hospital departments adhered well to the required annual standard benchmark set by the Danish Heart Failure Database (DHFD) audit commission to maintain one-year mortality at or less than 20% [23]. We found that the rate of one-year mortality in incident HF patients became even smaller during the three audit years. Another study reported that the annual national rates of one-year morality among incident HF Danish patients decreased to approximately 12.8% by 2010 [1] which is very close to the rate reported in this study. In Swedish patients, overall average annual one-year mortality rate was 22%, 21.3%, and 21.5% between 2007 and 2009. A previous Swedish study reported a similar one-year mortality of around 20% by 2000 [32]. A recent study reported similar rates while argued that 30-day and one-year mortality rates for Swedish patients after heart failure did not improve between the beginning of the new millennium and up to 2012 [33]. Nevertheless, two previous studies have concluded that survival for Swedish [33] and Danish [1] patients with HF has dramatically improved over the past three decades. This progress has been attributed to improvements in the quality of care in both countries [1, 32, 34].

Overall, literature has reported a wide range of mortality rates for patients with HF. For instance, one-year mortality (crude) rates for newly hospitalized patients with HF varied from 44.2% in a study in Scotland [35], and 33.1% in a Canadian study [36]. In Denmark, literature indicated that one-year mortality rate after first hospitalization decreased from 44% in 1983–1987 to nearly 33% between 2008 and 2012 [34]. Such country-specific rates seem higher than the Swedish rates. Still, the reported findings are not fully comparable since there would be differences in patient case-mix, case definition, as well as diagnostic and coding criteria. So, special consideration is needed when comparing mortality rates across different health care systems.

The same is true in our current study of mortality rates in the Swedish and Danish cohorts. First, each cohort has its own observation period. Second, one-year mortality for the Danish cohort has a specific denominator which is incident HF where more than half of the Danish patients were receiving care at outpatient departments [1]. In the Swedish cohort, we included patients with a first hospitalization for HF. Hence, severity of illness in the Danish cohort is much less than in Swedish patients.

The methodological approach we apply [12] represents a suitable tool for monitoring and profiling provider outcome of care. It combines single and multilevel regression models to evaluate the general, latent, unspecified organizational (i.e., department) effects which could condition patient survival over and above individual patient characteristics. Hence, we used the ICC [8, 10, 15] as well as the AUC values [12, 13, 16] to measure department observational effects. The analyses include two steps. The first step analyses patient level covariates in conventional single-level logistic regressions. The selection of these individual variables is based on the assumption that they are confounders (i.e., patient case-mix). The second step quantifies general contextual effects by measuring the ICC, and the increment in the AUC obtained by adding department level information (ID codes) into the corresponding MLRA model. By doing so, in both cohorts, the department variance (quantified with the ICC) was so small and the AUC analysis confirmed the interpretation of the ICC values. In the Swedish cohort, for each year, a single-level model with patient gender, age and risk score had a rather good discrimination ability. Similarly, in the Danish cohort, using only information on gender and age in single-level regression analyses gave a good AUC. However, considering the corresponding two-level (multilevel) models for both cohorts, adding the department level random effects did not improve the AUC value much. This demonstrates that knowledge of patient characteristics was enough to obtain a relatively high discriminatory accuracy.

To assess department general contextual effects (i.e., department performance), we interpreted these findings along with the mortality rates. We conclude that Danish departments had a homogeneously good (using DHFD benchmark) performance. This does not mean that monitoring hospitals using one-year mortality is no longer needed. The DHFD audit commission needs to continue to evaluate hospital (department) performance to assure future quality of care. In the Swedish cohort, department performance was also homogeneous. In reference to the findings for the Danish cohort, these mortality rates might express a higher basal risk for Swedish patients with HF. Fundamentally, Swedish departments could show a different performance for different observation periods, so, we confine our interpretation to the study period. Considering our empirical example, if the Swedish health authorities decided to improve one-year mortality after HF in this cohort, a comprehensive quality improvement strategy should target this group of patients in all departments. However, since individual level predictors (age, gender and the RS) revealed a high predictive accuracy, in such situations, a pragmatic strategy could focus on targeting high-risk patients across the hospital system.

In summary, with both cohorts in all observation periods, there is no point in ranking departments, considering the monitoring analysis for the Danish cohort and the profiling analyses for the Swedish cohort. Danish departments resemble random samples taken from a population of incident HF patients, while Swedish departments resemble random samples taken from a population of patients with the first hospitalization for HF.

### Methodological aspects

Despite having information on Swedish patient socio-economic factors and provider attributes, we did not include these variables in the models [5]. Our decision is supported by literature where it is recommended that profiling analyses should not remove differences in quality outcomes among providers that could be attributed to unfair or unequal delivery of care into specific social groups [5, 7, 20]. Further, inclusion of hospital attributes in *profiling* or quality monitoring models is highly debated in literature in light of the practical and methodological drawbacks considering potential endogeneity for these variables [5, 14]. Analogously, patient variables on episodes of care or certain clinical activities are not endorsed to be adjusted for in the profiling models [5, 7, 20].

Nevertheless, when needed, non-clinical patient characteristics and provider attributes could be investigated in complementary analyses [2], not the “*case-mix adjustment models*” [5], to identify best and poor practices (e.g., specialty care, staff-mix) and prioritize patient sub-groups (e.g., immigrants) who might be targeted with tailored health policies.

The obtained database for the Danish cohort had only basic information on patient age and gender and lacked information on other patient case-mix factors at admission. Adding more individual-level information (e.g., RS variable) for the Danish cohort would probably have further reduced the (already small) general effect of the department. That is, the minor general contextual effect of the department obtained in the multilevel model 2 is possibly an overestimation of the real department effect. Thus, there is no point to strive to explain more of something already very small. Literature supports this argument with a similar reasoning. Some studies concluded that similarities in clinical practices could result in small variance estimates [2, 37, 38]. Similarly, investigating postoperative, 30-day complications following pancreatic resection, Mehta et al [39] found a small variance (ICC = 4.2%) at the surgeon level and the hospital level (ICC = 1.7) in the unadjusted models. Hence, the authors decided not to further partition that small variance. Further, Ding et al [19] recommended that profiling analyses should be preceded by exploring the between-provider variability and if the provider variance is so small, there is no need for ranking or classifying providers. Of course, considering the Danish cohort, there is another alternative possibility that adding more patient-level covariates in the multilevel model 2 would not reduce the department variance at all. This situation has been reported repeatedly in literature including a study for Merlo et al [8]. Nevertheless, in the case we had the reverse scenario with a larger, meaningful variance for Danish departments, we would need to have more information on patient case-mix to accurately investigate (explain) such variation in performance in more details.

Essentially, instead of focusing on assessing the provider (department) variance in isolation, we consider that there is a multilevel continuum of patient variance that can be decomposed into between- and within-department components [8]. Therefore, the department variance is small when it is a small share of the total patient variance [2, 8, 12]. This idea is also expressed by the AUC based approach that we applied in this study [12]. There is a heavy debate about what should constitute a meaningful variation as there is no cut-off value for the ICC coefficient. Some researchers promoted a rule of thumb where the ICC should be at least around 10% [40] to warrant carrying out a multilevel analysis. On the other hand, some researchers such as Nezlek [41] and Hayes et al [42] argued that in the presence of data with a hierarchical nature, a multilevel modeling should be carried out, regardless of the size of variation between clusters. Yet, Hayes et al [42] admitted that an ICC of 5% is small. We believe that our approach provides a potential remedy for this dilemma. In our two-step approach, we jointly interpret the ICC along with the AUC value to indicate whether the between-department variance is meaningful or not. Conceptually, it is not feasible to specify a criterion for the amount of change in AUC value. However, we follow tradition in provider profiling literature on interpreting the AUC. For instance, an AUC value between 0.70 and 0.80 is considered as good and acceptable according to Bratzler [43], and Hosmer and Lemeshow [44, 45] among others [20, 46].

The AUC values were not very high in the individual, single-level models in both cohorts. So, there is a possibility that there might be some omitted individual predictors which might improve the models’ prediction of mortality by their addition. Even though, addition of such predictors would not change our interpretation, still, addition of department random effects into the models would not improve the obtained AUC [2, 12, 13]. This is also shown by the very small department variance which indicates homogeneous performance [2, 12]. Furthermore, in conventional literature, several authors such as O’Brien et al*[46],* Bratzler et al [43], Krumholz et al [20], Ash et al [14], and Normand et al [5] acknowledged that profiling models might not necessarily show very high AUC values. One of the dominant arguments here is that the aim of these evaluations is to assess provider outcomes of care (e.g., mortality, readmission) where a number of potential patient predictors (e.g., non-clinical characteristics, complications) have to be omitted from the models to produce proper profiling analyses [5, 7, 20]. Thus, some models had AUC values around 0.63 [20], yet the authors concluded that the analyses are still valid.

### Selected strengths and limitations

First, critiquing our data sources, in both countries, there are standardized quality control checks for data definition, acquisition and storage in clinical and population registers [1, 47]. We obtained data on patients admitted to clinical department at all hospitals in Denmark and Sweden as per our inclusion criteria. Data completeness on Swedish patients was optimal as we had access to full information on patient covariates included in this study. DHFD completeness is good (around 84%), covering the vast majority of the Danish patient population diagnosed with incident HF during the study period. Data on the one-year mortality indicator covers all patients who were diagnosed with incident HF upon their contact with a hospital during the study period, and who subsequently reported to DHFD. The majority of Danish departments have data completeness rates above 90% [1]. Thus, it could be wise to conduct a sensitivity analysis including missing patients who have not reported to DHFD.

Second, in our study, some departments had a smaller number of patients in comparison with other departments. Yet, estimation of variance is not affected, since in MLRA the small size departments (i.e., their residuals) are shrunken towards the overall mean to avoid statistical noise.

Third, in the Swedish cohort, the patient RS included a large number of patient case-mix variables (N = 36) with documented clinical relevance in literature [2, 21, 48, 49]. However, we stress that defining the best prediction models for profiling analyses that could be applied through different populations of HF patients and settings is beyond the scope of this study. Using available high quality data, we aimed to have parsimonious models yet not to be used for predicting future department performance [50], which is subject to continuous reforms along with iterative changes in practices. Therefore, model validation was less relevant considering the scope of this study.

Fourth, in general, our proposed analytical methodology can be deployed in different contexts, yet any attempt to generalize our findings should be done with great caution, as they concern the general context of Swedish and Danish hospital departments.

Fifth, in the Swedish cohort, the RS fulfils three main assumptions for a good case-mix adjuster [51]. Patient health condition at admission (variables used to develop the RS) are considered to be absent of *serious* endogeneity (e.g., not influenced by the provider or patient experience with the provider such as complications) [51]. Further, there is a linear effect for the RS as mortality increased with the increase in the RS [51]. Additionally, we tested the RS (centered, continuous variable) in a multilevel random slope analysis. We could have done either by specifying interaction terms for the RS with departments in a single-level model or by specifying a random slope in a multilevel model which is more parsimonious [15, 51, 52]. Hence, we specified a random slope for the RS by allowing the regression coefficient of RS to vary randomly at the department level. Yet the findings were not substantive, which indicating that this variable has a uniform effect across departments, which is a condition for a good case-mix adjuster [51, 53].

Sixth, we could develop a combined analysis that covers one period for each cohort instead of the separate analyses per year. However, we aimed to practically demonstrate the utility of our approach in performing annual profiling and monitoring analyses.

Finally, our results only concern the influence of the department on one-year mortality after HF during the study period. It is possible that the clinical departments show a larger effect for other outcomes and periods, or in other qualitative evaluations. In future research, we need to consider carrying out comprehensive assessments that cover longer periods of time and assess other quality indicators (e.g., medications, length of stay, readmission).

### Conclusion

Two sources of information are used to evaluate hospital department performance; the departments’ overall average of the patient one-year mortality, and the size of the general contextual effect (ICC and AUC). In our empirical examples, in all annual analyses, information on patient characteristics was the best predictor of one-year mortality risk, and this information did not improve by knowing where the patient was treated. Swedish and Danish hospital departments performed homogenously well, with a low one-year mortality rate after a diagnosis with heart failure. If health authorities decide to further reduce mortality rates considering the homogeneous performance of the departments, a comprehensive quality strategy should focus on all hospitals. Yet, a complementary assessment after 2007–2009 (for Swedish departments) and 2010–2013 (for Danish departments) is required to confirm whether department performance is still homogeneous or not in order to identify the most appropriate action.

## Ethics statement

### Swedish cohort

The Regional Ethics Review Board in southern Sweden (# 2012/637) as well as the data safety committees from the National Board of Health and Welfare and from Statistics Sweden approved the construction of the database.

### Danish cohort

Researchers applying for accessing anonymous data from the secretariat of the Danish Clinical Registries Program (RKKP) do not need special ethical approval by the data protection agency in Denmark. Access to DHFD data was granted after submission of a request to RKKP.
